# TWIST and ovarian cancer stem cells: implications for chemoresistance and metastasis

**DOI:** 10.18632/oncotarget.2428

**Published:** 2014-09-03

**Authors:** Sudhakar V. Nuti, Gil Mor, Peiyao Li, Gang Yin

**Affiliations:** ^1^ Department of Obstetrics, Gynecology and Reproductive Sciences, Yale School of Medicine, New Haven, CT, USA; ^2^ Department of Pathology, School of Basic Medicine, Central South University, Changsha, Hunan, China

**Keywords:** ovarian cancer stem cells, EMT, MET, TWIST1, chemoresistance

## Abstract

The transcription factor TWIST1 is a highly evolutionally conserved basic Helix-Loop-Helix (bHLH) transcription factor that functions as a master regulator of gastrulation and mesodermal development. Although TWIST1 was initially associated with embryo development, an increasing number of studies have shown TWIST1 role in the regulation of tissue homeostasis, primarily as a regulator of inflammation. More recently, TWIST1 has been found to be involved in the process of tumor metastasis through the regulation of Epithelial Mesenchymal Transition (EMT). The objective of this review is to examine the normal functions of TWIST1 and its role in tumor development, with a particular focus on ovarian cancer. We discuss the potential role of TWIST1 in the context of ovarian cancer stem cells and its influence in the process of tumor formation.

## INTRODUCTION

TWIST1 is a highly evolutionally conserved basic Helix-Loop-Helix (bHLH) transcriptional factor that functions as a master regulator of gastrulation and mesodermal development [1-4]. It has been implicated in the differentiation of multiple cell lineages, including muscle, cartilage, and osteogenic cells [5-7]. It has been shown that mice lacking TWIST1 died at E10.5, confirming its importance in development and differentiation[8]. Moreover, TWIST1 has been shown to be important in the regulation of programmed cell death and inflammation [4, 9], and it regulates genes that are essential for morphogenesis and cell migration [10].

In bHLH proteins, a basic DNA-binding domain is followed by two amphipathic α-helices separated by an inter-helical loop.[11] α-Helices are required for protein dimerization, which is a prerequisite for DNA-binding. The DNA-binding domain is able to recognize regulatory elements containing the hexanucleotide sequence (CANNTG), known as an E-box. The E-boxes are present in the regulatory elements of many lineage-specific genes, which accounts for the numerous pathways regulated by these transcription factors [12, 13]. bHLH proteins form either homodimers or heterodimers in the transcription process. The formation of a heterodimer plays a key role in regulating the transcriptional activity of a particular bHLH protein leading to an increase or decrease in its activity [14]. The bHLH transcription factors are traditionally classified into three subclasses: the ubiquitous Class A bHLH factors, which include HEB, E2-2 and E proteins (E12/E47); Class B, which comprises bHLH proteins that have specificity in tissue expression and form dimmers with class A molecules; and Class C, which do not form heterodimers with either class A or class B proteins [15]. The Twist proteins fall into a subfamily of the Class B bHLH factors and form heterodimers with E12 and E47 [16, 17]. Homo sapiens *TWIST1* gene is mapped to 7q21.2 and it contains two exons and one intron [18].

As indicated above, drosophila twist mutants fail to gastrulate, demonstrating its necessity for mesoderm differentiation and morphogenetic movement during gastrulation[19]. Subsequently, the important roles of *TWIST1* in development have been further elucidated by several studies [20, 21].

### TWIST1 and Normal Development

The discovery of *twist* lies in the investigation of morphogenesis. Simpson et al., building off of work by Lohs-Schardin and colleagues [22], who first isolated mutants of twist and discovered its effect on embryonic polarity and segmentation, found that the twist gene was involved in establishing the dorsoventral pattern of the Drosophila embryo [23]. Thereafter, Thisse et al. published a series of seminal papers further characterizing twist gene function in morphogenesis [1, 24]. Murre et al. proposed that twist encoded a basic helix loop helix (bHLH) transcription factor [25], which was subsequently confirmed by Wolf et al [11]. Recently, several groups reported on the human H-twist gene location, sequence, and its preliminary function in humans, and confirmed the findings from the animal studies showing that TWIST plays a role in embryonic development and that it encodes a bHLH protein that is 96% similar to the murine *M-twist* counterpart [26, 27]. In addition, it was shown that TWIST is highly expressed in embryos but not in adult tissues [28] and Doxorubicin can also affect the expression of TWIST to inhibit cellular differentiation pathways [29]. These early studies set the stage for its role in cancer stem cells and possibly drug resistance.

Given the essential role of the twist gene and TWIST transcription factor in development [20, 21], many abnormalities result when their functions go awry. In particular, germ-line mutations of the coding sequence of the *TWIST1* gene can cause many complications. The most prominent manifestation of such complications is craniosynostosis – the premature fusion of one or more of the sutures between the bones of the skull, as found in conditions such as Saethre-Chotzen syndrome [18, 30] and Baller-Gerold syndrome [31].

### TWIST1 as a Modulator of Inflammation

Inflammation is a critical biological process necessary for tissue repair, defense against microorganisms, and cell renewal. However, an uncontrolled inflammatory process could be detrimental for tissue homeostasis, leading to pathologic conditions, including cancer [32]. Consequently, the control of inflammation is critical to maintain the appropriate balance between defense of the organism and prevention of chronic inflammation. In addition to its role in embryogenesis, TWIST1 has been found to be a key regulator in the inflammatory processes. Initial studies have shown that TWIST1 is a major regulator of the NFκB signaling pathway [33]. TWIST1 has been shown to function as a modulator of NFκB by preventing the induction of pro-inflammatory cytokines [34, 35]. Sosic et al. first observed that upon treatment with tumor necrosis factor-alpha (TNFα), TWIST1 expression was associated with inhibition of cytokines by blocking the NFκB signaling pathway [36]. They proposed that TWIST1 regulates cytokine signaling by establishing a negative feedback loop that represses the NFκB-dependent cytokine pathway. Notably, the inflammatory pathway can also be affected by TWIST binding to NF-κB through a non-HLH mechanism [37]. Sharif et al. then reported the interaction between Type I interferons (IFNs), TWIST1, and the NFκB pathway [34]. Type I IFNs are pleiotropic cytokines and have immune regulatory functions by controlling the production of pro-inflammatory cytokines. The regulatory function of Type I IFNs has been shown in several patho-physiological settings such as delayed type hypersensitivity reactions, host defense to Listeria monocytogenes, and suppression of endotoxin-induced mortality [38] [39]. The mechanisms underlying the suppressive effects of Type I IFNs are not well understood but include the suppression of TNFα production. Sharif et al. found that Type I IFNs suppressed the production of TNFα through the regulation of the expression of the receptor tyrosine kinase Axl and downstream induction of TWIST1 [39]. TWIST1 binds to E-box elements in the TNFα promoter and suppresses NFκB-dependent transcription. Studies from our laboratory have shown that TWIST1 negatively regulates NFκB-dependent cytokine production through the regulation of has-miR-199a, which subsequently inhibits IKKβ and therefore NFκB activity [35]. The expression of IKKβ has been associated with the differential response to TNF-α [40]; therefore, regulation of IKKβ may switch the response of the TNFα from pro-inflammatory into pro-apoptotic or anti-inflammatory [41].

TWIST1 also plays an important role in regulating the function and differentiation of immune cells, particularly T helper 1 (Th1) cells. Niesner et al. showed that TWIST1 is expressed in Th1 effector memory (EM) cells, as it is induced by IL-12 via STAT4 and T cell receptor (TCR) signaling, which resulted in activation of Nuclear Factor of activated T-cells (NFAT) and NF-κB [42]. Interestingly, they found that expression of TWIST1 in Th1 lymphocytes limited the expression of the cytokines interferon-gamma (IFNγ), IL-2, and TNFα, and ameliorated Th1-mediated immunopathology in delayed-type hypersensitivity and antigen-induced arthritis. Pham et al. further elucidated the role of TWIST1 in Th1 cell development. They found that TWIST1 decreased IFN-γ production in Th1 cells by impairing the activity of the Th1 transcription factor network T-bet, STAT4, and Runx3 [43]. They also observed that TWIST1 is a key player in controlling – specifically limiting – both cell-mediated and humoral immunity [44]. In these studies, they were able to identify a feedback loop where cytokines, including IL-6, induced the STAT3-dependent expression of TWIST1 that repressed the transcription of the Il6ra, thereby limiting STAT3 activation and IL-6 responsiveness. The latter, in particular, limits the development of T follicular helper (Tfh) cells and T helper 17 (Th17) cells *in vivo*.

Another area of TWIST1's influence on inflammation is in adipose tissue [45]. Pettersson et al. described TWIST1's role in the inflammation of human white adipose tissue (WAT) by regulating at the transcriptional level the expression and secretion of inflammatory adipokines and fatty acid oxidation [46]. They also showed that TWIST1 levels are influenced by body weight status and weight loss [47]. Specifically, they found that low TWIST1 expression in human WAT was associated with obesity, insulin resistance, and increased secretion of inflammatory adipokines. They postulated that this effect may be due to an increased sensitively to TNFα's effects. All these results strongly support the critical role of TWIST1 maintaining the delicate balance during the inflammatory response and, furthermore, its importance in pathologic conditions associated with deregulation of inflammation, such as cancer.

### TWIST1 and Cancer

A growing number of studies have reported that TWIST1 is overexpressed in a variety of cancers, including breast cancer [48], gastric cancer [49, 50], colorectal carcinoma [15, 51], hepatocellular carcinoma [52, 53], prostate cancer [54, 55], bladder cancer [56, 57], nasopharyngeal carcinoma [58], head and neck squamous cell carcinoma [59, 60], esophageal cancer [61], endometrial cancer [62], and ovarian cancer [35, 41, 63, 64]. TWIST1's role in tumor formation and progression is an active area of research and its important role is becoming increasingly evident.

TWIST association with cancer has focused mainly on its potential role as a regulator of inflammation and in the generation of cancer cells with metastatic capacity. TWIST1 has been shown to play an important role in the process of Epithelial Mesenchymal Transition (EMT), which correlates with higher cancer aggressiveness and poor survival rates [65].

### Epithelial Mesenchymal Transition and the Process of Metastasis

The epithelial-mesenchymal transition (EMT) converts epithelial cancer cells into mesenchymal cancer cells with migratory capability and consequently the capacity to invade and metastasize. EMT is characterized by the loss of epithelial polarity and differentiation markers (such as E-cadherin and β-catenin) and the gain of mesenchymal markers (such as N-cadherin and vimentin) [66, 67]. Cancer cells with a mesenchymal phenotype are capable of migrating away from their primary tumor, interacting with stromal cells, invading their adjacent tissues, and intravasating into the lymphatic system and/or blood stream and settling into secondary tumor sites [68, 69].

As noted above, TWIST1 overexpression has been reported in a variety of cancers, and many observations have highlighted the role of TWIST1 in promoting cancer cell EMT and metastasis. Yang J et al. found that TWIST1 plays an important role in breast cancer EMT and metastasis [48]. They showed that suppression of TWIST1 by siRNA in highly metastatic mammary tumor cells specifically inhibits the cells’ ability to metastasize from the mammary gland to the lung. Ectopic expression of TWIST1 in the epithelial cancer cells results in loss of E-cadherin-mediated cell-cell adhesion, gain of mesenchymal markers, and induction of cell motility, implicating that Twist1 contributes to metastasis by promoting the expression of genes associated with the process of EMT. Yang Z et al. showed that TWIST1 regulates cell motility and invasion in gastric cancer cell lines, possibly through the N-cadherin and fibronectin production [50]. Luo GQ et al. then demonstrated that TWIST1 promotes the migration and invasion ability of gastric cancer cells through EMT [49]. Fan XJ et al. investigated the expression of TWIST1 and E-cadherin by immunohistochemistry in colorectal cancers [15]. They showed that expression of TWIST1 correlated with decreased membranous expression of E-cadherin. Importantly, TWIST1 up-regulation in the primary tumor had high correlation with the presence of metastatic tumors. Lee TK et al. revealed that TWIST1 overexpression correlated with hepatocellular carcinoma (HCC) metastasis, and high levels of TWIST1 mRNA level was found in metastatic HCC cell lines [52]. Further studies on the HCC cell lines showed that TWIST1 was able to induce EMT, which was correlated with increased HCC cell invasiveness [52]. Matsuo N et al. similarly found that TWIST1 induces a migratory effect in hepatocellular carcinoma by causing EMT [70].

Several studies have evaluated TWIST1 expression in primary tumors and revealed a correlation between TWIST1 expression and metastatic disease. Kwok WK, et al. found that TWIST1 was highly expressed in a majority of prostate cancer patients, and TWIST1 expression levels were positively correlated with Gleason grading and metastasis, suggesting its major role in the progression of prostate cancer [54]. Zhang Z, et al. showed that TWIST1 expression was higher in bladder cancer tissues compared with nonmalignant tissues and that increased TWIST1 expression levels were correlated with high grade and advanced stage tumors, indicating its role in the in the development and progression of bladder cancer [57]. In addition, they showed that TWIST1 was significantly higher in the metastatic lesions compared with the primary site, and the increased TWIST1 expression in bladder cancer was associated with decreased expression of E-cadherin. Shen CH et al. showed that TWIST1 may act upstream of E-cadherin, which can regulate the expression of beta-catenin, providing further evidence that EMT factors TWIST1, E-cadherin, and beta-catenin play important roles in the metastatic progression of bladder cancer [56]. Horikawa T et al. suggested that the induction of TWIST1 directly contributes to the metastatic nature of nasopharyngeal carcinoma [59]. Yu L et al. (2012) revealed that the overexpression of TWIST1 plays an important role in the metastasis of hypopharyngeal tumors [60]. Since TWIST1 correlated with EMT and c-fos and MMP-9 expression, they concluded that the TWIST/c-fos/MMP-9 pathway might play an important role in the metastasis of cells.

Yang MH et al. showed that TWIST1 is directly regulated by HIF-1α [71]. Under hypoxic conditions, HIF-1α is stabilized and promotes EMT and metastatic phenotypes through the regulation of TWIST1 by binding directly to the hypoxia-response element (HRE) in the TWIST1 proximal promoter. In another seminal paper, Yang MH et al. showed that TWIST1 regulates the polycomb-group protein Bmi1 and that both TWIST1 and Bmi1 were mutually essential to promote EMT and repress expression of E-cadherin and p16INK4a in head and neck cancers, with the up-regulation of both proteins conferring the worst prognosis for patients [72]. In addition, Cheng et al. showed the activation of STAT3 by Il-6 induced *TWIST1* expression at both the protein and mRNA levels, and thereby promoting metastasis [4].

### Chemoresistance and Angiogenesis

Chemoresistance is a major limitation on the successful treatment of all types of cancers. Therefore, identification of the molecular mechanisms associated with chemoresistance is a critical step toward preventing recurrent disease, the clinical outcome associated with the survival of cancer cells to chemotherapy [73]. Emerging evidence suggests that TWIST1 plays an important role in the chemoresistance of cancer cells. Wang et al. found that up regulation of TWIST1 was responsible for the development of acquired paclitaxel-resistance in nasopharyngeal carcinoma cells, and ectopic expression of TWIST1 led to increased resistance to microtubule-disrupting agents, including paclitaxel and vincristine [74]. Zhang X et al. suggested that the TWIST1-induced taxol resistance is mediated through protection against apoptosis and TWIST1-mediated taxol resistance may be regulated through its positive involvement with the Akt pathway [58]. Pham CG et al. found that TWIST1 plays an important role in NF-κB-dependent chemoresistance [75].

In addition to inducing chemoresistant properties, TWIST1 also has a role in cancer angiogenesis. Mironchik et al. showed that stable over-expression of TWIST1 in breast cancer cells increased VEGF synthesis [76]. Furthermore, *in vivo* tumors with over-expressed TWIST1 exhibited higher vascular volume and vascular permeability. Niu et al. then showed that the high expression of TWIST1 in HCC was associated with hgher microvessel density and an up-regulation of VEGF and N-cadherin [53]. Thus, TWIST1 induces angiogenesis in many cancers.

### TWIST1 and Ovarian Cancer

One of the first reports linking TWIST1 and ovarian cancer was the study from Kajiyama and colleagues [77]. They found that TWIST1 expression predicts poor clinical outcomes in patients with clear cell carcinoma (CCC) of the ovary, suggesting that TWIST1 may play a critical role in the progression of CCC. They proposed that TWIST1 expression in the tumor could be used as a potentially prognostic indicator. Hosono et al. further evaluated the value of TWIST1 as a prognostic marker in patients with epithelial ovarian carcinoma (EOC) and reached a similar conclusion as that reported by Kajiyama et al.[63]. They found that positive TWIST1 expression significantly predicted poorer progression-free survival and overall survival and that in a multivariate analysis, it was the only independent prognostic factor for survival.

Since then, several studies have been conducted to elucidate the function of TWIST1 in ovarian carcinomas. Two main areas of TWIST1 influence have emerged – metastasis and chemoresistance. As will be discussed below, cancer stem cells – in this case ovarian cancer stem cells – play a large part in both cancer progression and chemoresistance. We will discuss first the broader findings on the role of TWIST1 in ovarian cancer and then we will review the role of TWIST1 in ovarian cancer stem cells.

As described for other solid tumors, TWIST1 is thought to play an important role in the metastatic process of ovarian cancer. Terauchi et al. showed that there was a correlation between TWIST1 expression and epithelial ovarian cancer (EOC) cellular morphology [64]. Specifically, they showed that the suppression of TWIST1 expression in these cells alters the cellular morphology from a mesenchymal, fibroblastic, motile phenotype to an epithelial phenotype while concurrently inhibiting the adhesion of these cells to mesothelial monolayers. This was one of the first indications of the role of Twist in the multistep EMT process of peritoneal metastasis dissemination in ovarian cancer. Yoshida et al. then showed in ovarian surface epithelium (OSE) tumors that TWIST1 expression increased step-wise in benign, borderline, and malignant tumors [78]. Elloul et al. similarly found that TWIST1 expression was significantly higher in solid ovarian cancer metastases compared to primary carcinomas and effusions[79]. Wang et al. explored TWIST1's regulation of E-cadherin showing that ovarian carcinomas, compared with normal ovary tissues, had a greater abundance of TWIST1 expression and a decrease in E-cadherin expression [80]. When Twist expression was silenced, however, E-cadherin expression then increased. As we have seen in other cancers, the Twist-E-cadherin axis of metastasis may be implicated in ovarian cancer as well.

While the control of metastasis is one area of TWIST1's involvement in ovarian cancer, it is also involved in another important phenomenon – chemoresistance. One of the first papers that chronicled the effects of TWIST1 in ovarian cancer was by Wang et al., who showed that TWIST1 expression was associated with taxol resistance in ovarian cancer cell lines, indicating a possible larger role in the “development of resistance to certain microtubule-disrupting agents in human cancer” [74]. Additional studies were conducted by Li et al., who demonstrated that TWIST1 was highly upregulated in cisplatin-resistant ovarian cancer cells as compared to cisplatin-sensitive cells [81]. Similarly, we found a significant increase of Twist expression in an animal model of recurrence. Tumors from recurrent disease after treatment with Paclitaxel had higher levels of TWIST1 as well as of genes associated with mesenchymal differentiation (Slug, vimentin) and stemness (ALDH1, KLF4 MyD88) [73]

### Ovarian Cancer Stem Cells

Hematopoietic cancers and solid tumors contain a population of cells that possess the capacity to self-renew and to produce the heterogeneous lineage of cancer cells that comprise the whole tumor [82, 83]. These cells have been defined as cancer stem cells (CSCs) and are characterized by unique plasticity and differentiation potential. Current evidence suggests that CSCs are the putative mediators of chemoresistance and tumor progression [84-86]. It is thought that CSCs are able to survive conventional chemotherapeutic treatments, which usually target fast-dividing cells and give rise to recurrent tumors that are more chemoresistant and more aggressive [87-89].

Using CD44 as a marker, our group identified Epithelial Ovarian Cancer (EOC) stem cells based on their capacity to recreate the original tumor when injected into mice [90-94]. We have fully characterized the molecular phenotype of these cells [90, 91, 95] and demonstrated their plasticity – they are able to differentiate and lose stemness markers *in vitro* and *in vivo* and, more importantly, are able to differentiate into endothelial cells [91, 96]. Indeed, we find that the presence of these cells is associated with shorter progression-free survival in EOC patients [94]. Our findings are in line with other studies that have shown the existence of tumor-initiating cells (TICs) in EOC through the use of different markers suggestive of the heterogeneity of the disease [90, 97-101]. Nevertheless, there is a consensus that CD44+ EOC cells represent the chemoresistant phenotype [97-99, 102-104].

Our most recent data identified an additional differentiation potential in the EOC stem cells. As explained in more detail below, by undergoing EMT, these cells acquire markers and behavioral attributes of mesenchymal cells, including the capacity to form compact spheroids and increased migratory and invasive capacity [105]. This suggests that the EOC stem cells are the source of metastatic disease.

### Spread of Ovarian Cancer

**Figure 1:**
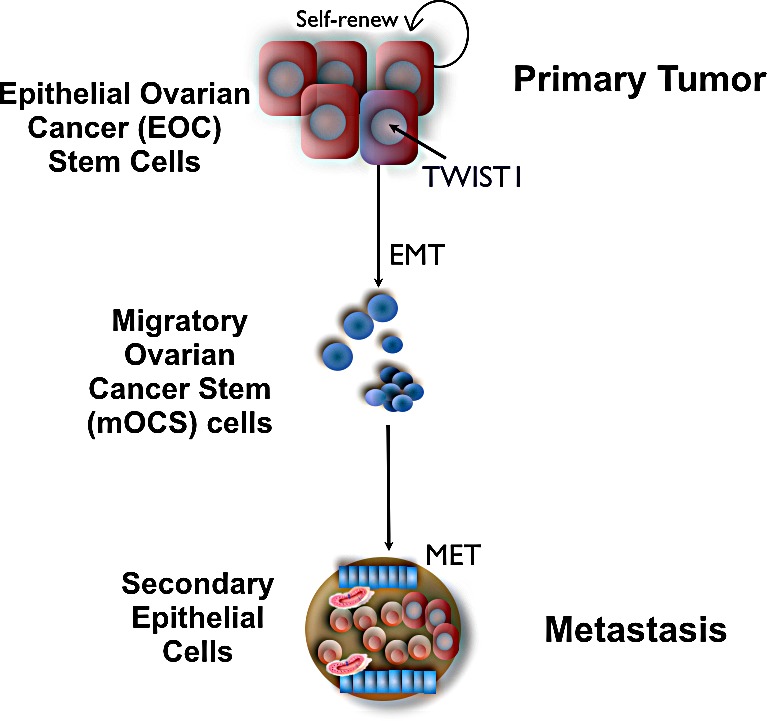
Role of TWIST1 on Epithelial Mesenchymal Transition of Epithelial Ovarian Cancer Stem cells TWIST1 expression in epithelial ovarian cancer stem cells promotes their differentiation into mesenchymal cells with cancer stem cells properties and migratory capacity. Once attached to a different region of the peritoneal cavity these cells undergo MET and establish a metastatic tumor with epithelial characteristics.

Metastatic ovarian cancer is characterized by carcinomatosis in the abdomen and pelvis. It usually spreads locally and rarely through the circulation [106, 107]. This suggests that ovarian cancer cells are able to lose cell-to-cell contact and shed into the peritoneal cavity to establish metastatic disease [108]. As usually observed in ascitic fluid from ovarian cancer patients, cancer cells in the peritoneal cavity often aggregate and form spheroid-like structures, which can subsequently implant throughout the abdomen with a varying extent of peritoneal invasion [109]. Our studies have shown that the EOC stem cells represent the cell population that can differentiate into the spheroid-forming cells with metastatic potential. We have been able to recreate in vitro, from a pure population of EOC stem cells the process of differentiation from epithelial cells to mesenchymal spheroid cells (m-spheroid cells) similar to those observed in the ascites [35, 105]. Moreover, we showed that m-spheroid cells can revert back to an epithelial phenotype through the mesenchymal-epithelial transition (MET) and give rise to epithelial cultures that have lost stemness markers [105]. We hypothesized that a subpopulation of EOC stem cells have the capacity to undergo EMT, acquire the capacity to migrate and invade out of the solid tumor to generate m-spheroid cells, and establish metastatic disease (Fig. 1).

### Epithelial-Mesenchymal Transition and Metastasis in Ovarian Cancer

Epithelial cells are characterized by the propensity for cell-cell adhesion, planar and apical-basal polarity and lack of mobility [34, 35]. As stated above, EMT is a process by which epithelial cells lose these characteristics and acquire mesenchymal properties, including the capacity to migrate and invade [110]. Thus, in cancer, EMT has been associated with metastasis [111]. There are multiple factors inducing EMT in human solid tumors including, but not limited to, inflammation, hypoxia, fibrosis, necrosis, apoptosis, senescence, and DNA damage [111-114]. Therefore, it is not possible to define a canonical EMT pathway; neither has it been possible to define the target cells, which have the capacity to respond to these signals and undergo transformation.

Epithelial ovarian cancer stem cells maintain the epithelial characteristics of epithelial tumors; however, they lack the capacity of migration and therefore metastasis [105]. We hypothesized that within the cancer stem cells, a small fraction of cells could acquire the potential for migration and tumor metastasis – “the metastatic ovarian cancer stem cells”. Recently, we described, in addition to the epithelial ovarian cancer stem cells and the EOC cells, the characterization of a third type of ovarian cancer cells with migratory capacity: migratory ovarian cancer stem cells (mOCS cells) (Fig 1) [105]. Most importantly, the differentiation of EOC stem cells with epithelial phenotype to these mOCSs with mesenchymal characteristics is associated with the up-regulation of the transcription factor TWIST1 [35]. The following novel insights exemplify the importance and potential link between cancer stem cells and TWIST1 in the progression of ovarian cancer, and serve as a framework for understanding the role of TWIST1 in ovarian cancer.

Studies from our lab have demonstrated that TWIST1 is a major regulator of EOC ‘stemness’ by controlling stem cell differentiation through the positive regulation of miRNAs miR-199a and miR-214 [41] [35]. These miRNAs are inhibitors of PTEN and IKKβ, therefore controlling cell proliferation, apoptosis, and inflammation. All these aspects are important for maintaining the characteristics of cancer stem cells [41]. Interestingly, TWIST1 expression is tightly controlled in EOC stem cells in order to maintain their epithelial phenotype. We found that EOC stem cells have an active mechanism to prevent the accumulation of TWIST1 protein even in the presence of high levels of mRNA, which involves the ubiquitin-proteasome system. However, when the levels of TWIST1 protein increase, we found that EOC stem cells differentiate into the migratory ovarian cancer stem cells (mOSC cells) with mesenchymal characteristics [105]. While TWIST1 was constitutively degraded in EOC stem cells, E12 expression stabilized TWIST1 protein expression. The expression of both TWIST1 and E12 were induced by hypoxia/HIF-1, yet the lack of TWIST1 in EOC stem cells in the presence of hypoxia prevented EMT, which illuminates the essential role of TWIST1 in the regulation of ovarian cancer differentiation.

Kajiyama et al. established an ovarian cancer cell line that is resistant to paclitaxel and found that these cells displayed high levels TWIST1 protein and had a greater migratory and metastatic potential compared to cells that are paclitaxel sensitive [115]. Latifi et al. also observed that cisplatin induced EMT in ovarian cancer cells, where TWIST1 expression was significantly increased in response to the chemotherapy and was associated with increased migration of the cells [116].

**Figure 2:**
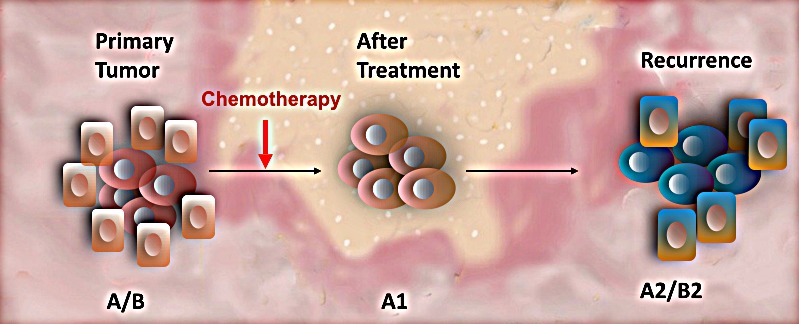
Effect of Chemotherapy on Cancer Stem Cells Primary tumors are heterogeneous made up by a hierarchy of cancer cells (A/B). Conventional chemotherapy targets the fast dividing cancer cells (B) leaving the chemo-resistant cancer stem cells (A). However, the cells composing the residual tumors revealed different molecular characteristics than those observed on the original cancer stem cells (A1). Consequently, the secondary tumor is different from the primary disease and will respond differently to therapy.

Thus, based on these studies and our own, we could postulate that chemo-resistant cells undergo EMT in response to chemotherapy as result of up-regulation of TWIST1 expression. Indeed, we recently demonstrated that Paclitaxel treatment can induce molecular modifications on EOC stem cells enhancing the acquisition of mesenchymal characteristics while maintaining their stemness potential [73]. Our findings suggest that chemotherapy does not only enriches for putative EOC stem cells, but the treatment can also induce specific phenotypic modifications in the surviving EOC stem cells (Fig. 2). These changes might contribute to recurrence where the cells are more aggressive and also show a higher degree of chemoresistance. Using *in vitro* and *in vivo* models we found the expression of TWIST1 and other EMT genes in the EOC stem cells that survive Paclitaxel treatment. These molecular changes might provide the original EOC stem cells the capacity to migrate and establish multiple metastatic sites, a characteristic of recurrent disease [73].

Moving forward, more studies are needed to characterize the ovarian cancer stem cells before and after chemotherapy and the functionality of TWIST1 in each of these cell cohorts to understand how it functions in different cellular environments to created better targeted therapies to destroy the cancer and prevent recurrence.

## CONCLUSION

Although initially identified as a transcription factor associated with embryonic development, TWIST1 may play additional roles in the regulation of tissue homeostasis. Furthermore, its role in cancer opens a field of opportunities for the development of new diagnostic tests and therapeutic modalities, with a particular focus on ovarian cancer stem cells.
